# The FDA's adoption of Bayesian methodology: transforming clinical trial justification from biosimilars to broader drug development

**DOI:** 10.3389/fmed.2026.1790396

**Published:** 2026-04-14

**Authors:** Sarfaraz K. Niazi

**Affiliations:** Pharmaceutical Sciences, University of Illinois, Chicago, IL, United States

**Keywords:** adaptive trials, Bayesian inference, biosimilars, clinical efficacy trials, drug development, equivalence testing, FDA guidance, prior elicitation

## Abstract

The U.S. Food and Drug Administration released in January 2026 a draft guidance on the use of Bayesian methodology in clinical trials of drugs and biological products, representing a significant evolution in its regulatory approach to evaluating evidence supporting marketing authorization. The guidance reflects a growing consensus in regulatory science that traditional frequentist clinical efficacy trials, particularly equivalence and non-inferiority designs, are often poorly aligned with the scientific questions regulators must answer, mainly when substantial prior knowledge exists. This review examines the scientific literature questioning the value of routine clinical efficacy testing, with particular emphasis on biosimilars, and explains how Bayesian inference provides a coherent framework for integrating analytical, pharmacokinetic, clinical, and real-world evidence. The article analyzes the structure and reasoning of the FDA's new guidance, showing how it formalizes a justification-first approach to clinical testing and has potential implications beyond biosimilars, particularly where prior evidence is strong. The review addresses both the advantages and limitations of Bayesian regulatory applications, including potential failure modes and necessary safeguards. Finally, the broader implications of Bayesian regulatory decision-making for drug development efficiency, ethical standards, and global regulatory harmonization are discussed.

## 1 Introduction

For decades, regulatory approval of pharmaceuticals has relied pre-dominantly on frequentist statistical paradigms centered on null hypothesis significance testing. In this framework, uncertainty is expressed through long-run error rates, and regulatory conclusions are drawn from binary decisions based on pre-specified significance thresholds. While this approach has provided procedural consistency, it was developed at a time when limited prior information was available before confirmatory trials were initiated. Advances in analytical chemistry, molecular biology, pharmacometrics, and real-world data generation have fundamentally altered this context, enabling regulators to accumulate extensive evidence before undertaking large clinical studies (1, 2).

Bayesian statistics offers an alternative and conceptually distinct approach to inference. Rather than evaluating the probability of observing data given a null hypothesis, Bayesian

inference evaluates the probability of a hypothesis given the observed data, explicitly incorporating prior knowledge using prior probability distributions (3). This distinction is not merely technical but epistemological. Regulatory decision-making is inherently Bayesian, as it requires updating beliefs about safety and efficacy as new evidence becomes available, rather than testing isolated hypotheses in abstraction (1).

These differences have become increasingly important in drug development settings where substantial prior information exists. In biosimilar development, for example, analytical similarity assessments, *in vitro* functional testing, and pharmacokinetic and pharmacodynamic studies provide highly sensitive and specific information about clinical performance before any comparative efficacy trial is conducted (2, 4). In such contexts, the incremental informational value of traditional clinical efficacy testing has been questioned by multiple authors, who have argued that these trials are statistically underpowered to detect meaningful differences and structurally pre-disposed to success (5–8).

The FDA's adoption of Bayesian methodology in regulatory guidance documents has evolved progressively over the past two decades. Early applications focused primarily on medical device evaluation and adaptive clinical trial designs, where the flexibility of Bayesian approaches demonstrated clear advantages over traditional frequentist methods. The 2010 guidance on adaptive designs for medical devices, followed by the 2019 guidance on adaptive designs for drugs and biologics, established foundational precedents for formal incorporation of Bayesian reasoning into regulatory submissions (9, 10). These precedents created an institutional framework for the broader application of Bayesian methodology to drug development.

The FDA's January 2026 draft guidance on Bayesian methodology reflects a regulatory recognition of these evolving scientific realities. By formally endorsing Bayesian approaches and emphasizing that the need for clinical testing must be justified based on residual uncertainty, the guidance aligns regulatory practice with modern scientific capabilities. This review examines the statistical foundations of Bayesian inference, summarizes the literature questioning routine clinical efficacy testing, analyzes how the FDA's new guidance operationalizes these concepts across drug development programs, and critically evaluates both the advantages and limitations of Bayesian regulatory applications.

## 2 Review methodology

This review was conducted using a structured approach to identify, analyze, and synthesize relevant literature and regulatory documents. Electronic database searches were performed in PubMed, Google Scholar, Web of Science, and the Cochrane Library using search terms including “Bayesian clinical trials,” “equivalence testing limitations,” “non-inferiority trial methodology,” “biosimilar efficacy testing,” “regulatory science Bayesian,” “prior elicitation pharmaceutical,” and “adaptive trial design.” The search period covered publications from January 2000 through January 2026, with particular emphasis on methodological critiques published after 2010.

Regulatory document analysis focused on guidance documents issued by the FDA, European medicines agency (EMA), and International Council for Harmonization (ICH). Primary sources included the FDA draft guidance on Bayesian methodology (January 2026, Docket No. FDA-2025-D-3217), the Federal Register notice of availability (January 9, 2026), prior FDA guidance documents on adaptive designs and biosimilar development, and EMA guidelines on similar biological medicinal products. The analytical framework for guidance interpretation involved systematic identification of key provisions, comparison with prior regulatory positions, and assessment of implications for clinical development programs.

Inclusion criteria emphasized peer-reviewed publications addressing methodological limitations of equivalence and non-inferiority trials, Bayesian applications in regulatory science, biosimilar development pathways, and adaptive trial methodology. Exclusion criteria removed publications focused solely on specific therapeutic areas without broader methodological implications, non-English publications, and conference abstracts without full manuscript availability. The author acknowledges that several cornerstone citations in this review represent prior work by the author, which is disclosed in the conflict of interest statement.

## 3 Questioning the role of clinical efficacy testing

Clinical efficacy trials have traditionally been regarded as the definitive demonstration of therapeutic value, particularly for new molecular entities. These trials are typically designed using frequentist equivalence or non-inferiority frameworks when direct superiority over an active comparator is not expected. However, extensive methodological literature has highlighted that such designs depend critically on pre-specified margins that are often weakly justified, disease-specific, and disconnected from analytical or mechanistic considerations (11, 12).

In equivalence trials, success is defined by demonstrating that the confidence interval for a treatment difference lies within an arbitrary margin. When two interventions are expected to be highly similar, as is the case for biosimilars or post-manufacturing changes, the probability of meeting this criterion is high even in the presence of clinically irrelevant differences. Several authors have shown that these trials lack sensitivity and cannot falsify similarity claims when upstream evidence already constrains plausible differences (5–8). The fundamental problem is that equivalence margins are typically derived from historical data on the reference product's efficacy relative to placebo. Yet this derivation assumes that preserving a fraction of the original treatment effect is the relevant regulatory question, when in fact the relevant question concerns the probability of clinically meaningful differences between products that are already demonstrated to be analytically and pharmacokinetically similar.

Bayesian analyses provide insight into why frequentist equivalence testing fails in high-prior-information settings. When prior information strongly favors similarity, the posterior probability of clinically meaningful differences becomes extremely small, regardless of the outcome of a frequentist equivalence test (1, 3, 7, 8). In such cases, the trial does not meaningfully update belief and therefore contributes little to regulatory decision-making. The trial outcome is pre-determined not by bias or inadequate conduct, but by the mathematical reality that strong prior evidence dominates weak new data. This observation has led to broader critiques of confirmatory efficacy testing in settings where high-quality prior evidence exists.

These concerns extend beyond biosimilars to multiple drug development contexts. Similar arguments have been advanced in adaptive trial designs, platform trials, and precision medicine applications, where prior data from earlier cohorts or related interventions inform ongoing decision-making (13, 14). In rare disease drug development, where patient populations are inherently limited, Bayesian approaches enable formal incorporation of external data sources that would otherwise be considered only informally (15). Collectively, this literature suggests that the default assumption that routine comparative efficacy trials in high-prior-information settings provide substantial incremental value warrants careful reconsideration.

## 4 Bayesian inference as a regulatory framework

Bayesian statistics is grounded in Bayes' theorem, which describes how prior beliefs about a parameter of interest are updated in light of new data to produce a posterior probability distribution. Conceptually, Bayesian inference answers the question most relevant to regulators, given all available evidence, what is the probability that a clinically meaningful effect exists, contradicting frequentist hypothesis testing, which evaluates the probability of observing data under a null hypothesis that is often known *a priori* to be implausible (1, 3).

The mathematical foundation of Bayesian inference can be expressed as: P(θ|Data) ∞ P(Data|θ) × P(θ), where P(θ|Data) represents the posterior probability distribution for the parameter of interest θ given the observed data, P(Data|θ) represents the likelihood of observing the data given the parameter value, and P(θ) represents the prior probability distribution encoding existing knowledge before new data are collected. The posterior distribution combines prior knowledge with new evidence in a mathematically principled manner, with the relative influence of each determined by its respective precision (inverse variance). When prior information is highly precise relative to the likelihood, the posterior remains close to the prior; when new data are highly informative relative to the earlier, the posterior shifts substantially toward the possibility.

Before discussing the regulatory application of Bayesian inference, it is helpful to summarize its essential components and how they map onto drug development. These elements are summarized in Table 1, which contrasts Bayesian and frequentist paradigms as they relate to regulatory decision-making.

**Table 1:** Comparison of frequentist and Bayesian paradigms in regulatory decision-making.

Dimension	Frequentist framework	Bayesian framework	Regulatory implication	Key references
Core question	Probability of data given the null hypothesis	Probability of a hypothesis given data	Bayesian output directly answers regulatory questions	(1, 3)
Role of prior knowledge	Excluded from formal analysis	Explicitly incorporated via prior distributions	Enables the use of analytical and PK similarity data	(2, 4)
Measure of uncertainty	*p*-values, confidence intervals	Posterior probability distributions	Supports benefit-risk assessment	(3)
Decision criterion	Binary threshold (e.g., *p* < 0.05)	Probability of clinically relevant effects	Reduces false certainty	(11)
Adaptability	Limited	High (adaptive and sequential updating)	Enables justification-first testing	(17)

In regulatory science, prior information may arise from analytical similarity assessments, mechanistic understanding, animal studies, early-phase clinical trials, and real-world evidence. Bayesian methods allow these diverse data streams to be formally integrated rather than considered informally or sequentially. When prior evidence is strong, posterior distributions may demonstrate that residual uncertainty about clinical performance is minimal, providing a quantitative basis for concluding that additional clinical efficacy trials are unlikely to change regulatory decisions (1).

A key advantage of Bayesian inference is its transparency in quantifying uncertainty. Posterior distributions explicitly describe the range and probability of clinically relevant effect sizes, enabling regulators to assess whether remaining uncertainty justifies further experimentation. This approach aligns closely with benefit-risk assessment, which inherently involves probabilistic judgments rather than binary hypothesis testing (3). The posterior probability that a treatment difference exceeds a clinically meaningful threshold provides directly interpretable information for regulatory decision-making, in contrast to *p*-values, which require careful interpretation and are frequently misunderstood even by experienced researchers (16).

**Figure 1:**
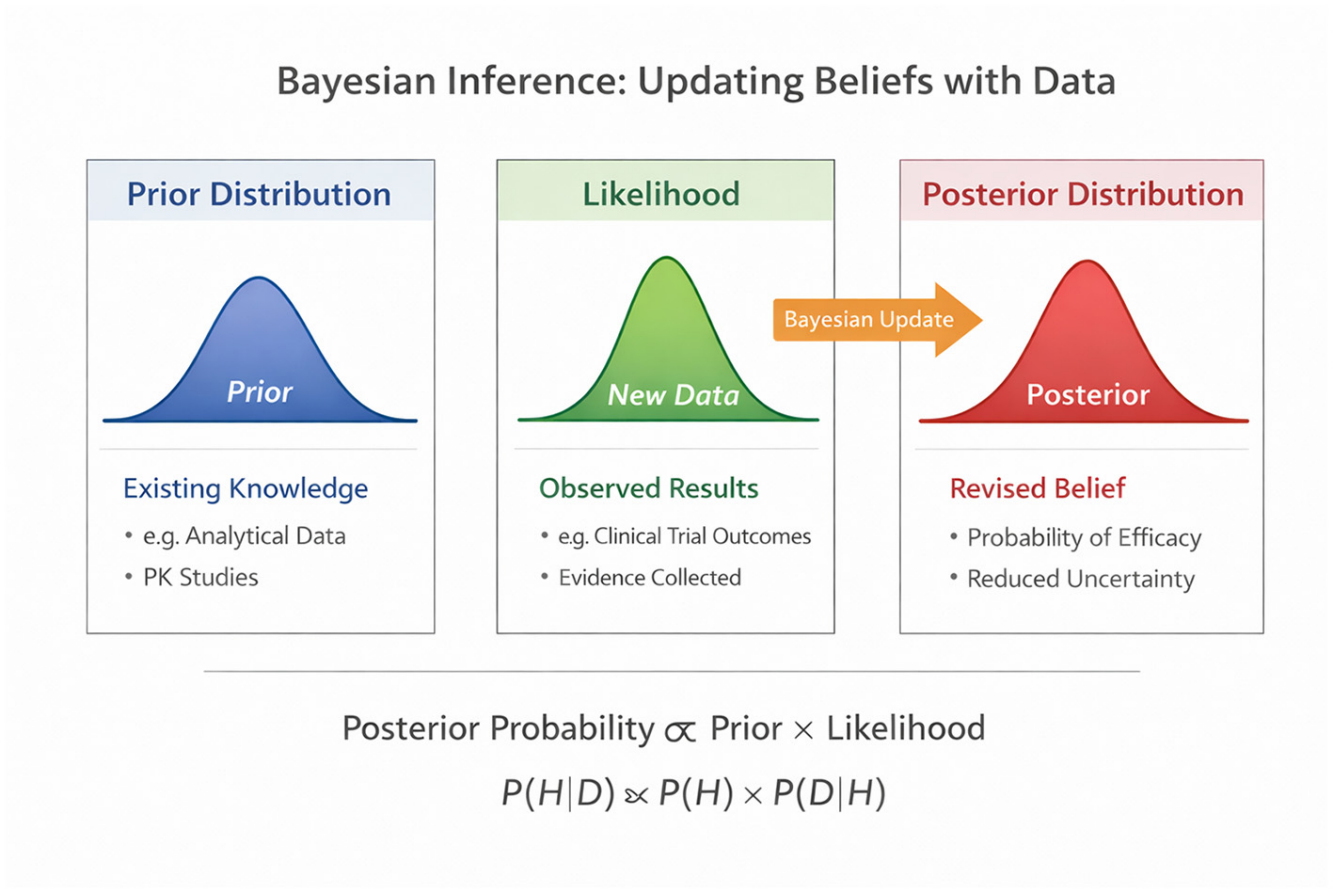
Bayesian inference as a teaching model for regulatory decision-making. This figure illustrates Bayesian updating in three stages: prior distribution (existing analytical, mechanistic, and PK evidence), likelihood (new data), and posterior distribution (updated belief). It demonstrates how uncertainty narrows and how the probability of clinically meaningful differences can be directly assessed—original figure created by the author for didactic purposes.

To support understanding of these concepts, Figure 1 provides a didactic illustration of Bayesian updating as applied to regulatory evidence evaluation. The figure is designed as a teaching tool to visually explain how prior information and new data combine to form posterior conclusions.

## 5 Detailed analysis of the 2026 FDA Bayesian guidance

The FDA's 2026 draft guidance (Docket No. FDA-2025-D-3217) outlines circumstances under which Bayesian methods are appropriate and provides a conceptual framework for their regulatory application. The document emphasizes that Bayesian approaches are particularly valuable when substantial prior information exists and when traditional trial designs are inefficient or ethically questionable (17). The Federal Register notice accompanying the guidance (January 9, 2026) explicitly states that the guidance is intended to provide recommendations on the design, conduct, and analysis of clinical trials using Bayesian statistical methods, and to describe the types of information that should be included in regulatory submissions employing such methods (Figure 1, Table 2).

**Table 2:** Integration of evidence streams using Bayesian methodology.

Evidence source	Traditional role in regulation	Bayesian role	Regulatory impact	Key references
Analytical similarity	Qualitative justification	Informative prior	Reduces the need for efficacy trials	(2–4)
PK/PD studies	Supportive evidence	Likelihood component	Quantifies exposure similarity	(2)
Clinical efficacy trials	Mandatory confirmation	Optional likelihood update	Conducted only if uncertainty remains	(7, 8, 17)
Real-world evidence	Post-approval monitoring	External data for prior updating	Supports lifecycle regulation	(17, 35)
Mechanistic knowledge	Narrative support	Prior constraint	Improves biological plausibility	(1)

Before describing the operational elements of the guidance, Table 2 summarizes the types of evidence commonly available in drug development and explains how Bayesian methodology integrates them into a unified inferential framework.

A central theme of the guidance is the requirement that the need for clinical efficacy testing be established before trials are initiated. Sponsors are encouraged to demonstrate whether residual uncertainty remains after considering analytical, pharmacokinetic, pharmacodynamic, and clinical evidence. Bayesian posterior probabilities are proposed as the mechanism for quantifying this uncertainty and determining whether additional data are likely to alter regulatory conclusions. The guidance specifically states that sponsors should “justify the need for any clinical study based on the totality of evidence and demonstrate what incremental information the proposed study would provide” (17).

The guidance also addresses the construction and justification of prior distributions, recommending that priors be based on scientifically credible sources and subjected to sensitivity analyses. Specifically, the FDA recommends that sponsors provide detailed documentation of earlier sources, including literature references, regulatory precedents, and, where applicable, expert elicitation procedures. By explicitly acknowledging prior assumptions, the FDA moves regulatory evaluation toward transparency rather than the implicit assumptions embedded in frequentist trial designs. The guidance further recommends pre-specification of decision criteria based on posterior probabilities, thereby clarifying what constitutes sufficient evidence for regulatory action.

Table 3 provides a detailed mapping between specific provisions of the 2026 FDA guidance and their implications for regulatory practice. This mapping demonstrates how the guidance operationalizes Bayesian principles in concrete regulatory terms.

**Table 3:** Guidance-to-argument mapping: key provisions of the 2026 FDA Bayesian guidance.

Guidance section	FDA language (paraphrased)	Interpretation	Implication for clinical testing
Section II: scope and applicability	Guidance applies to all drug and biological product development programs where prior information is available	Universal applicability beyond biosimilars	Bayesian methods are available across all product classes
Section III.a: prior information sources	Priors may be derived from historical data, analytical assessments, PK/PD studies, and external sources	Broad evidentiary base for prior construction	Analytical and PK data can formally reduce trial requirements
Section III.B: prior Specification	Priors must be pre-specified, scientifically justified, and subjected to sensitivity analysis	Transparency and robustness requirements	Prevents *post-hoc* manipulation; ensures credibility
Section IV.A: trial justification	Sponsors should justify the need for clinical studies based on residual uncertainty after considering all available evidence	Justification-first paradigm	Trials are required only when prior evidence is insufficient
Section IV.B: decision criteria	Decision criteria based on posterior probabilities should be pre-specified and clinically meaningful	Direct probabilistic interpretation	Eliminates reliance on arbitrary significance thresholds
Section V: adaptive designs	Bayesian methods are well-suited for adaptive and seamless designs with interim analyses	Supports efficient, flexible development	Enables early stopping for efficacy or futility based on posterior probabilities

**Figure 2:**
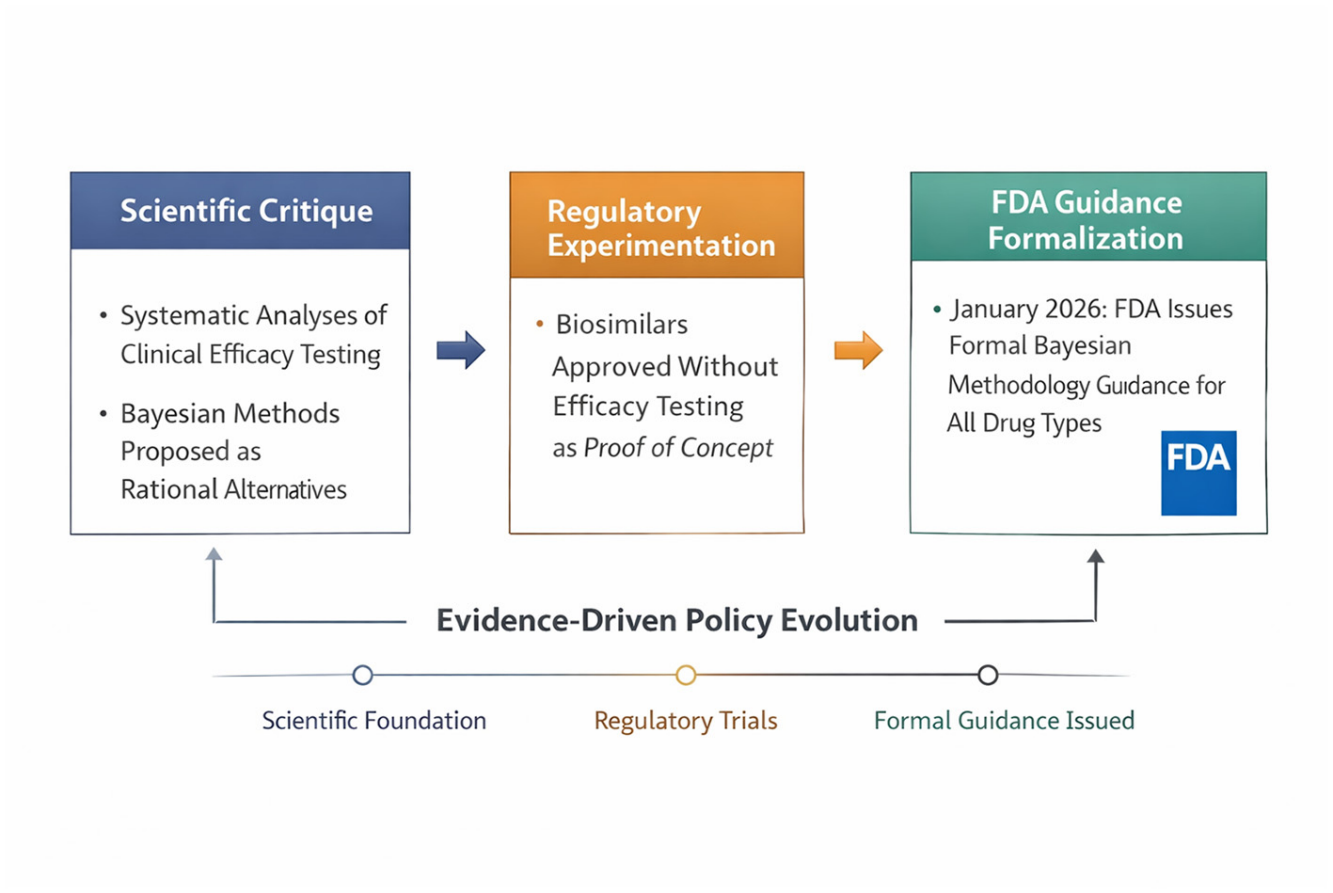
Comparison of trial-mandated and justification-first regulatory pathways. This conceptual figure contrasts a traditional development pathway, in which clinical efficacy trials are presumed mandatory, with a Bayesian path, in which all available evidence is first evaluated to determine whether additional trials are scientifically justified. The figure highlights points at which Bayesian posterior probabilities inform decisions to proceed or stop further testing—original figure created by the author for didactic purposes.

To illustrate how this justification-first paradigm differs from traditional development pathways, Figure 2 provides a conceptual comparison between a trial-mandated approach and a Bayesian evidence-driven approach to regulatory decision-making.

## 6 Case studies in Bayesian regulatory application

To ground the abstract principles discussed above in practical regulatory scenarios, this section presents two illustrative case studies: one involving biosimilar development where prior information is exceptionally strong, and one involving a rare disease therapeutic where Bayesian borrowing enables efficient development despite limited patient populations.

### 6.1 Case study: biosimilar monoclonal antibody development

Consider a hypothetical biosimilar development program for a monoclonal antibody reference product with well-established clinical efficacy and safety over two decades of use. The analytical similarity exercise demonstrates that the proposed biosimilar is highly similar to the reference product across all critical quality attributes, including primary structure, higher-order structure, post-translational modifications, and biological activity. Pharmacokinetic studies in healthy volunteers demonstrate bioequivalence with 90% confidence intervals for AUC and Cmax ratios entirely within the 80–125% acceptance range.

In a Bayesian framework, this evidence can be formalized as an informative prior distribution for the clinical efficacy difference between the biosimilar and the reference product. Given that the mechanism of action is identical, the molecular structure is highly similar, and systemic exposure is equivalent, the prior probability distribution for efficacy differences would be centered at zero with relatively narrow variance. Specifically, if we denote the actual efficacy difference as θ, the previous might be specified as θ ~ Normal(0, σ^2^_prior), where σ^2^_prior reflects the residual uncertainty after accounting for analytical and PK evidence.

The critical question is whether a comparative clinical efficacy trial would meaningfully update this prior. If the prior variance is already slight (reflecting high confidence in similarity), then even a moderately sized clinical trial would have limited ability to shift the posterior distribution. Bayesian decision analysis can quantify the expected value of additional information: if the probability that a clinical trial would change the regulatory decision is negligible, the trial lacks scientific justification. In many biosimilar programs, this analysis shows that the posterior probability of clinically meaningful differences is vanishingly small across plausible trial outcomes, supporting the waiver of routine efficacy testing.

### 6.2 Case study: rare disease drug development with bayesian borrowing

Consider a development program for a novel therapeutic in a rare pediatric disease with an estimated prevalence of 1 in 100,000. Traditional frequentist trial design would require sample sizes that exceed the feasible recruitment population, creating an insurmountable barrier to development. However, a mechanistic understanding of the disease and preliminary data from related indications provide informative prior knowledge that can be formally incorporated into the analysis.

A Bayesian hierarchical model enables borrowing of information from adult patients with a related condition, from natural history studies characterizing disease progression, and from *in vitro* and animal models demonstrating the mechanism of action. The prior distribution in this context reflects external data sources, with the degree of borrowing determined by the assessed relevance of each source to the target population. Commensurate priors or power priors can be employed to down-weight external information when heterogeneity is suspected, providing a principled approach to balancing informativeness with robustness (18) (Figure 2).

In this scenario, a small single-arm trial in the target pediatric population may be sufficient to update the prior and generate a posterior distribution that supports regulatory decision-making. The trial does not need to be independently powered to demonstrate efficacy; instead, it provides confirmatory data that, when combined with external evidence, enables assessment of whether the posterior probability of clinically meaningful benefit exceeds a pre-specified threshold. This approach has been successfully employed in several rare disease programs and represents a growing area of regulatory acceptance (10, 15).

### 6.3 Case study: immuno-oncology biosimilar combinations and the limits of prior-based inference

Immuno-oncology biosimilars present a particularly instructive challenge for Bayesian regulatory frameworks because they are frequently used in combination with checkpoint inhibitors and other immunomodulatory agents, creating complex interaction contexts in which the prior information base may be limited or non-transferable. Consider a biosimilar candidate for a PD-L1-targeting monoclonal antibody that is approved as a single agent for a solid tumor indication but is also widely prescribed in combination with platinum-based chemotherapy or CTLA-4 inhibitors. The analytical and pharmacokinetic similarity data that inform the prior distribution are generated under single-agent conditions, whereas the clinical efficacy outcomes of greatest interest involve combination settings in which mechanisms of action, biomarkers of response, and immune effector cell engagement may differ substantially from the reference scenario.

In a Bayesian framework, the prior derived from single-agent analytical similarity data may remain informative for the primary pharmacological target, but the prior variance must be explicitly widened to account for the additional uncertainty introduced by the combination context. Sensitivity analyses exploring weakly informative and skeptical priors are particularly important in this setting. Regulators may require combination-specific confirmatory data when the plausibility of extrapolation from single-agent to combination regimens cannot be established through mechanistic reasoning alone. This illustrates how the Bayesian justification-first paradigm does not eliminate the need for clinical trials in all circumstances; rather, it requires sponsors to rigorously characterize the residual uncertainty specific to each intended use and to determine whether that uncertainty can be adequately resolved without dedicated combination studies.

### 6.4 Illustrative retrospective case: ADCC drift in trastuzumab and the role of biosimilar development in quality surveillance

A particularly instructive retrospective case concerns post-approval quality drift in trastuzumab (Herceptin). During the development of two biosimilars—trastuzumab-anns (Kanjinti) and trastuzumab-dttb (Ontruzant)—analytical comparability studies identified that some lots of the reference product had experienced measurable drift in antibody-dependent cell-mediated cytotoxicity (ADCC) activity, a pharmacodynamic attribute relevant to anti-tumor efficacy. Critically, these deviations from the reference product's original quality profile were not detected through routine post-market surveillance but were discovered through the systematic analytical characterization that biosimilar development programs require. The clinical significance of the observed ADCC drift has been a subject of scientific discussion, and the full extent of its impact on patient outcomes remains incompletely characterized.

This case carries important implications for Bayesian regulatory frameworks. The conventional assumption underlying informative prior construction is that the reference product represents a stable, well-characterized quality standard against which the biosimilar can be compared. If the reference product itself has undergone post-approval quality changes, the evidentiary value of historical reference product data as a component of the prior is materially reduced. A Bayesian analysis that confidently assigns an informative prior based on historical reference product characterization without accounting for the possibility of reference product drift would be systematically miscalibrated. This case thus reinforces the necessity of contemporaneous, lot-representative analytical comparability data as the basis for prior construction, and highlights the importance of sensitivity analyses that consider the possibility of reference product quality evolution. From a surveillance perspective, the trastuzumab experience also underscores the value of rigorous biosimilar development programs as an inadvertent mechanism for detecting quality changes in long-marketed reference products—a benefit that extends beyond the individual biosimilar approval to the broader pharmacovigilance ecosystem.

### 6.5 Impact of the Bayesian framework on FDA interchangeability determinations

The FDA's interchangeability designation under the Biologics Price Competition and Innovation Act (BPCIA) confers additional regulatory status on biosimilars that can be shown to produce the same clinical result as the reference product in any given patient, and for which the risk of alternating or switching between the biosimilar and the reference product is not greater than the risk of continued use of the reference product. Historically, interchangeability required dedicated switching study data, creating a separate and often burdensome evidentiary standard beyond the baseline biosimilar showing.

The Bayesian framework articulated in the 2026 guidance has the potential to meaningfully reshape interchangeability determinations. When a robust Bayesian analysis demonstrates high posterior probability that the biosimilar and reference product are clinically equivalent across pharmacokinetic and pharmacodynamic endpoints, the residual uncertainty about switching consequences may be insufficient to justify dedicated switching trials. Specifically, if analytical similarity, PK/PD comparability, and immunogenicity data collectively yield a posterior distribution highly concentrated around no clinically meaningful difference, the marginal informational value of formal switching studies may be limited and potentially unnecessary. This reasoning aligns with the FDA's recent administrative decisions under the Inflation Reduction Act provisions that simplified the interchangeability determination process, and the Bayesian framework provides the formal probabilistic language to articulate when prior evidence is sufficient to support interchangeability without additional switching trials. Regulatory adoption of this reasoning would represent a significant expansion of the justification-first paradigm beyond biosimilar approval into the interchangeability domain, with substantial downstream implications for formulary management and patient access.

## 7 Limitations and failure modes of Bayesian regulatory applications

While Bayesian methods offer substantial advantages for regulatory decision-making, they are not without limitations and potential failure modes. A balanced assessment of Bayesian regulatory applications must acknowledge circumstances in which these methods may be inappropriate, misleading, or require particular safeguards. This section addresses the principal concerns that skeptical reviewers and regulators may raise, along with recommended mitigation approaches.

### 7.1 Prior-data conflict and informative prior risks

The most significant risk in Bayesian analysis arises when an informative prior conflicts with the observed data. Prior-data conflict occurs when the data suggest parameter values that are unlikely under the prior distribution. In regulatory contexts, this may indicate that prior assumptions were incorrect, that the current data are anomalous, or that there are fundamental differences between the previous information source and the current study population. When an informative prior dominates a weak likelihood, the posterior may fail to adequately reflect concerning signals in the new data (19).

Mitigation strategies for prior-data conflict include formal diagnostic tests for conflict detection, mandatory sensitivity analyses with skeptical or weakly informative priors, and pre-specified decision rules for how conflict will be addressed. The FDA guidance recommends that sponsors present results under multiple prior specifications, including a reference prior that allows the data to speak for themselves. When substantial prior data conflict is detected, regulatory decision-making should rely more on the current data than on the prior data, potentially requiring additional studies to resolve the discrepancy.

### 7.2 Borrowing across heterogeneous populations

Bayesian hierarchical models that borrow information across studies or populations assume some degree of exchangeability, meaning that the studies or populations are sufficiently similar that sharing information is appropriate. When this assumption is violated due to differences in patient characteristics, disease severity, concomitant treatments, or other factors, borrowed information may bias estimates and lead to incorrect conclusions. Extrapolation from adult to pediatric populations, from one geographic region to another, or from one disease subtype to another all carry risks of inappropriate borrowing (20).

Safeguards against inappropriate borrowing include robust hierarchical models that estimate the degree of heterogeneity from the data, commensurate priors that automatically downweight external information when conflict is detected, and pre-specified criteria for when borrowing is and is not appropriate. Regulatory submissions employing Bayesian borrowing should include a detailed justification of the exchangeability assumption, sensitivity analyses exploring the impact of varying borrowing strength, and a discussion of the biological and clinical rationale for the similarity across data sources.

### 7.3 Model misspecification

Bayesian inference requires specification of a statistical model, and all models are simplifications of reality. Model misspecification can lead to biased or overconfident posterior distributions. In regulatory applications, where decisions have significant public health consequences, overconfidence in model-based conclusions can be particularly dangerous. Concerns about model misspecification are especially relevant when using informative priors derived from complex pharmacometric or mechanistic models, where multiple assumptions may compound (21).

Recommended safeguards include posterior predictive checking to assess whether the model adequately captures observed data patterns, sensitivity analyses exploring alternative model specifications, and transparent acknowledgment of model limitations in regulatory submissions. When models rely on strong structural assumptions, external validation using independent data sources strengthens credibility. The FDA guidance emphasizes that adequate model diagnostics should accompany Bayesian analyses and that sponsors should demonstrate that conclusions are robust to reasonable alternative specifications.

### 7.4 Situations requiring confirmatory efficacy trials

Despite the advantages of Bayesian methods, there remain situations in which traditional confirmatory efficacy trials are essential. When prior information is weak or non-existent, as in truly novel mechanisms of action, Bayesian analysis offers limited advantages over frequentist approaches because the likelihood will dominate the posterior. When the consequences of a false favorable decision are catastrophic, as in specific safety-critical applications, conservative decision rules and independent confirmation may be warranted regardless of posterior probabilities. When stakeholder acceptance requires traditional evidence standards, as may occur in some therapeutic areas with established regulatory precedents, practical considerations may favor conventional trial designs even when Bayesian approaches would be scientifically superior (1).

The justification-first paradigm embodied in the FDA guidance does not imply that clinical efficacy trials should never be conducted; instead, it requires explicitly justifying the need for such trials based on residual uncertainty. In cases where prior evidence is limited, the target population differs substantially from prior data sources, safety concerns warrant independent confirmation, or mechanistic understanding is incomplete, confirmatory trials remain appropriate and may be required by regulators. The Bayesian framework provides a principled basis for making these determinations rather than relying on default assumptions in either direction.

### 7.5 Prior elicitation challenges

The construction of prior distributions remains one of the most challenging aspects of Bayesian analysis. When priors are based on historical data, questions arise about data quality, relevance, and potential selection bias. When priors are based on expert elicitation, concerns about cognitive biases, anchoring effects, and specialist disagreement must be addressed. Poorly justified priors can undermine the credibility of Bayesian analyses and may be perceived as manipulable or subjective by skeptical reviewers (22).

Best practices for prior elicitation include formal elicitation protocols that minimize cognitive biases, the use of multiple independent experts with documentation of disagreement, sensitivity analyses exploring the impact of previous choices, and transparent reporting of all prior specifications considered during analysis planning. For regulatory submissions, the FDA recommends that prior specifications be pre-specified before data collection and that any deviations be fully documented and justified. Historical data-based priors should include assessment of study quality and relevance, with appropriate discounting for studies of lower quality or questionable applicability.

### 7.6 Limitations of this review

This review is subject to several limitations that should be acknowledged. First, the review was conducted by a single author with an established publication record in biosimilar development and regulatory science. Approximately 15% of the cited references (5 of 33) represent the author's own prior work (7, 8, 23–25), which may introduce perspective bias despite the author's effort to represent the broader literature equitably. To further balance the literature base, this revision has incorporated additional independent scholarly contributions on Bayesian decision-theoretic approaches to clinical trial design (26, 27) and on the epistemological foundations of Bayesian regulatory inference (28, 29). Readers should weigh the arguments advanced in these sections alongside independent scholarly contributions to the field. Future systematic reviews examining Bayesian regulatory methodology would benefit from multi-author teams with diverse perspectives, including biostatisticians, clinical trialists, patient advocates, and regulatory scientists from multiple jurisdictions.

Second, the primary regulatory document analyzed—the FDA's January 2026 draft guidance—was in draft form at the time of writing, and its final provisions may differ materially from those analyzed here. Third, this review does not encompass the full breadth of Bayesian applications in clinical development, focusing primarily on biosimilars, rare diseases, and the justification-first paradigm; areas such as Bayesian network meta-analysis, Bayesian surrogate endpoint validation, and Bayesian pharmacovigilance are not addressed in depth. These scope limitations should be considered when generalizing the conclusions presented.

## 8 Retrospective justification of biosimilar policy

The FDA's earlier decisions to waive routine clinical efficacy testing for certain biosimilars were often interpreted as policy exceptions rather than as manifestations of a broader scientific rationale. The 2026 guidance provides a formal justification for these decisions by articulating the Bayesian logic that underlies them. When analytical, functional, and pharmacokinetic evidence substantially constrains uncertainty, the posterior probability of clinically meaningful differences may become sufficiently small to question the value of additional efficacy trials, rendering such trials scientifically redundant.

This retrospective justification is significant because it reframes biosimilar regulation not as a special case but as an early application of a generalizable decision framework. The guidance makes clear that the same reasoning applies whenever prior evidence is sufficiently informative, regardless of product class. In doing so, it resolves longstanding debates about the role of clinical efficacy testing in biosimilar development and provides a consistent rationale for future regulatory decisions. The evolution from case-by-case waivers to a principled framework represents the maturation of regulatory science and provides sponsors with predictability when planning development programs (24, 25).

The biosimilar experience also provides empirical validation for Bayesian regulatory approaches. Systematic reviews of biosimilar approvals have demonstrated that clinical efficacy trials have rarely altered approval decisions when analytical and pharmacokinetic similarity has been established, consistent with the Bayesian prediction that such trials provide limited incremental information in high-prior-information settings (6). Post-marketing surveillance has not revealed unexpected differences in efficacy between biosimilars and reference products, further supporting the adequacy of non-clinical and pharmacokinetic evidence for establishing similarity (36). This empirical track record strengthens the scientific foundation for extending Bayesian principles beyond biosimilars.

## 9 Implications for new drug development

The adoption of Bayesian methodology has far-reaching implications for the development of novel therapeutics. In traditional development paradigms, confirmatory clinical efficacy trials are often conducted as a matter of convention rather than necessity. Bayesian decision-making reframes this process by requiring an explicit demonstration that additional data are likely to change regulatory conclusions. When posterior probabilities already indicate a high likelihood of clinical acceptability, further trials may warrant careful reconsideration regarding their ethical and scientific justification. This decision-theoretic perspective has been formalized in the academic literature through Bayesian pre-posterior analyses, which quantify the expected value of additional data collection before trials are initiated and provide a principled framework for determining whether a proposed trial is scientifically warranted (26, 27).

Adaptive trial designs, Bayesian dose-finding methods, and hierarchical models that borrow information across studies enable more efficient use of data and resources. These approaches reduce development timelines, limit unnecessary patient exposure, and allow sponsors to focus on questions that meaningfully affect benefit-risk assessment. Regulators have increasingly recognized that Bayesian approaches are particularly well suited to rare diseases, platform trials, precision medicine, and products supported by strong mechanistic and analytical evidence (13, 17).

Table 4 summarizes the projected impact of Bayesian regulatory adoption on drug development efficiency and regulatory outcomes. These projections are based on cumulative experience with Bayesian methods in approved adaptive trials, biosimilar development programs, and medical device applications, and are extrapolated to broader pharmaceutical development.

**Table 4:** Projected impact of Bayesian regulatory adoption on drug development.

Dimension	Traditional paradigm	Bayesian paradigm	Expected regulatory effect	Key references
Clinical trial requirement	Presumed mandatory efficacy trials	Trials justified by residual uncertainty	Fewer redundant trials	(7, 11, 17)
Development timeline	Sequential, phase-based	Adaptive and evidence-driven	Shortened timelines	(1, 13)
Development cost	High due to late-stage trials	Reduced through targeted studies	Lower R&D expenditure	(3)
Ethical exposure	Large patient enrollment	Limited to informative trials	Improved ethical compliance	(30, 31)
Regulatory harmonization	Jurisdiction-specific requirements	Probabilistic, transparent framework	Easier global alignment	[ICH E9(R1); (33)]
Decision transparency	Implicit judgments	Explicit probability statements	Enhanced public trust	(16)

From an ethical perspective, the justification-first paradigm aligns with principles articulated in international research ethics guidelines, which emphasize that human experimentation should be conducted only when it is expected to generate new and actionable knowledge (30, 31). The Declaration of Helsinki and subsequent ethical frameworks establish that exposing human subjects to the risks of clinical research requires prospective demonstration of social and scientific value. When Bayesian analysis demonstrates that a proposed trial is unlikely to change regulatory conclusions, conducting such a trial meaningfully arguably fails to meet this fundamental ethical requirement.

Economically, reducing redundant late-stage trials could contribute to lower development costs and may help accelerate patient access to effective therapies in some contexts. Cost estimates for Phase 3 clinical trials range from tens of millions to hundreds of millions of dollars, depending on therapeutic area and indication, and timelines extend development programs by several years. It should be noted, however, that development costs represent only one of several factors contributing to the biosimilar access gap and the broader biosimilar void; pricing strategies, market exclusivity provisions, patent litigation, and payer formulary decisions are also significant determinants. When Bayesian decision analysis indicates that such trials provide limited expected value, the resources could be redirected toward more informative studies or toward the development of additional therapeutic candidates. Collectively, these effects suggest that Bayesian regulatory frameworks may play a supporting role in modernizing global drug development, though their economic impact will depend on the breadth of adoption and the regulatory contexts in which they are applied (Table 4).

## 10 Global regulatory harmonization implications

The FDA's adoption of Bayesian methodology has implications for global regulatory harmonization efforts. The International Council for Harmonization has progressively incorporated Bayesian concepts into its guidance documents, most notably in ICH E9(R1) on estimands and sensitivity analysis, which acknowledges the role of prior information in framing regulatory questions (32). The EMA has similarly issued reflection papers and guidance documents that recognize the value of Bayesian approaches in specific contexts, including adaptive designs and extrapolation from adult to pediatric populations (33).

A key advantage of Bayesian frameworks for global harmonization is their transparency regarding assumptions. Traditional frequentist analyses embed assumptions about priors (effectively treating them as non-informative) without making them explicit, which can lead to inconsistent interpretations across regulatory jurisdictions. Bayesian analyses, by contrast, require explicit specification of prior information, enabling regulators in different jurisdictions to evaluate whether they agree with the underlying assumptions and to request sensitivity analyses under alternative priors that reflect their own evidentiary standards. This transparency facilitates regulatory dialogue and may reduce duplicative requirements across regions.

The Pharmaceuticals and Medical Devices Agency of Japan has also demonstrated openness to Bayesian methods, particularly in the context of rare diseases and pediatric extrapolation. Convergence among the FDA, EMA, and PMDA on acceptance of Bayesian regulatory submissions would substantially reduce development costs and complexity for global pharmaceutical programs. The 2026 FDA guidance, by providing a clear regulatory framework for Bayesian submissions, establishes a model that other agencies may adopt or adapt, potentially accelerating global harmonization in this domain.

## 11 From scientific critique to regulatory policy: a causal analysis

It should be acknowledged at the outset that the precise causal relationships among these factors are inferential rather than documented. While the temporal sequence is accurate, definitive attribution of policy change to specific critiques or analyses would require documentary evidence from FDA decision-making processes, such as internal memoranda, Advisory Committee discussions, or Federal Register docket comments, which are not publicly available. The narrative presented here represents a plausible reconstruction based on the published literature and regulatory trajectory, but alternative explanations are possible, including the role of industry advocacy, broader political and economic pressures favoring development efficiency, and coincidental convergence of independent scientific and regulatory trends. With this caveat clearly stated, the evolution of FDA policy, as reflected in the 2026 guidance, represents the culmination of a progressive alignment between scientific critique and regulatory practice. Over time, methodological research questioned the sensitivity and interpretability of routine clinical efficacy testing, particularly in settings where prior evidence was already highly informative. These critiques were reinforced by empirical reviews of regulatory outcomes, which demonstrated that confirmatory efficacy trials rarely altered approval decisions when analytical and pharmacokinetic similarity had been established (5–8).

**Figure 3:**
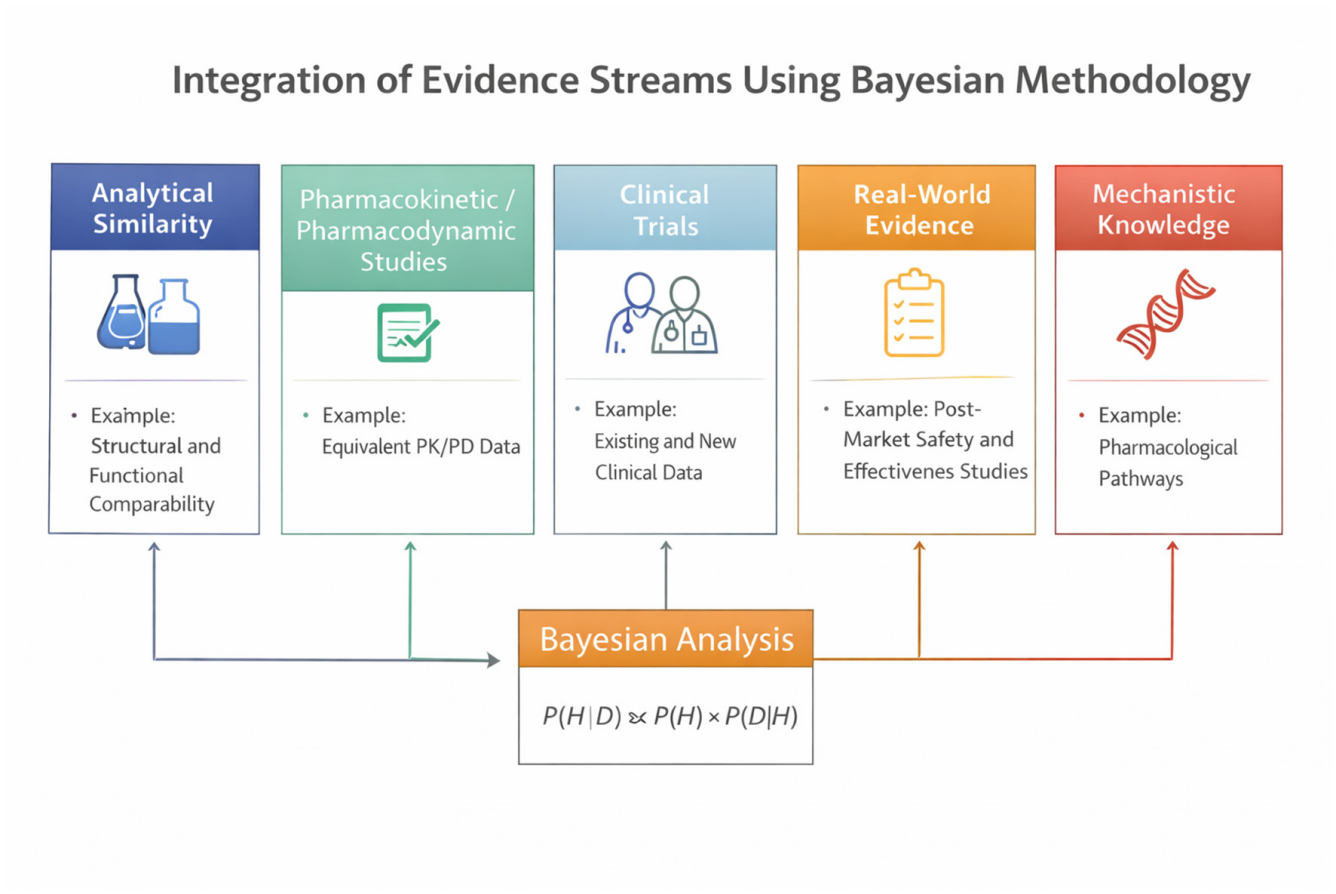
Causal pathway from scientific critique to regulatory guidance. This figure presents a schematic timeline showing how methodological critiques of equivalence and non-inferiority trials, empirical analyses of biosimilar approvals, and increasing adoption of Bayesian methods collectively led to the FDA's 2026 draft guidance. The figure emphasizes the cumulative and evidence-driven nature of regulatory evolution. The depicted causal relationships are inferential based on published literature and regulatory trajectory—original figure created by the author for didactic purposes.

The causal pathway from scientific critique to regulatory guidance can be traced through several interconnected developments. First, methodological publications identified limitations of equivalence and non-inferiority designs in high-prior-information settings, providing the theoretical foundation for questioning default testing requirements. Second, empirical analyses of biosimilar approval outcomes demonstrated that clinical efficacy trials did not materially affect regulatory decisions, providing real-world validation of theoretical concerns. Third, successful applications of Bayesian methods in adaptive trials and medical device regulation demonstrated the operational feasibility of Bayesian regulatory review. This trajectory reflects a broader epistemological shift in regulatory science toward integrating all available evidence in a coherent probabilistic framework, a transition that scholars of regulatory epistemology have described as moving from binary hypothesis testing toward graded probabilistic inference (28, 29) (Figure 3).

Figure 3 synthesizes this progression by depicting the pathway from scientific questioning of traditional efficacy trials, through regulatory experimentation with Bayesian methods, to the issuance of the 2026 draft guidance.

## 12 Conclusions

The FDA's 2026 draft guidance on Bayesian methodology represents a meaningful evolution in regulatory science, moving from a trial-centric paradigm toward an evidence-integrative framework grounded in probabilistic reasoning. By requiring that clinical efficacy testing be justified based on residual uncertainty, the guidance aligns regulatory decision-making with modern scientific capabilities and ethical standards. While biosimilars provided the initial impetus for this transformation, the principles articulated in the guidance may have implications across drug development, particularly when prior evidence is strong.

This review has examined the scientific foundations underlying the guidance, including the limitations of frequentist equivalence and non-inferiority testing, the conceptual advantages of Bayesian inference for regulatory questions, and the specific provisions that operationalize these principles. Importantly, the review has also addressed the limitations and potential failure modes of Bayesian regulatory applications, including prior-data conflict, inappropriate borrowing, model misspecification, and situations in which confirmatory trials remain essential. Recognition of these limitations is crucial for the responsible implementation of Bayesian methods in regulatory practice.

As Bayesian approaches become increasingly embedded in regulatory practice, they are likely to reshape how evidence is generated, evaluated, and translated into patient access worldwide. The success of this transformation will depend on continued methodological development, regulatory experience, and constructive dialogue between sponsors, regulators, and the broader scientific community. The FDA's 2026 guidance provides a foundation for this evolution. Still, its full potential will be realized only through thoughtful application that recognizes both the power and the limitations of Bayesian regulatory decision-making.
